# A high offset stem design does not increase stem migration under full weight bearing in cementless total hip arthroplasty: a model-based RSA study

**DOI:** 10.1186/s42836-024-00290-y

**Published:** 2025-02-04

**Authors:** Tobias Reiner, Robert Sonntag, Jan Philippe Kretzer, Michael Clarius, Eike Jakubowitz, Stefan Weiss, Stefan Kinkel, Tilman Walker, Tobias Gotterbarm, Timo Albert Nees

**Affiliations:** 1 Department of Orthopaedics, Heidelberg University Hospital, Schlierbacher Landstraße 200a, Heidelberg, 69118 Germany; 2 Department of Orthopaedics, Laboratory of Biomechanics and Implant Research, Heidelberg University Hospital, Schlierbacher Landstr. 200a, Heidelberg, 69118 Germany; 3 Department for Orthopedics and Traumatology, Vulpius Klinik GmbH, Vulpiusstrasse 29, Bad Rappenau, 74906 Germany; 4 Department of Orthopaedic Surgery, Laboratory for Biomechanics and Biomaterials, Hannover Medical School, Anna-Von-Borries-Str. 1-7, Hannover, 30625 Germany; 5 ARCUS Clinics Pforzheim, Rastatter Str. 17-19, Pforzheim, 75179 Germany; 6 Department for Orthopedics and Traumatology, Kepler University Hospital GmbH, Johannes Kepler University Linz, Krankenhausstrasse 9, 4020 Linz and Altenberger Strasse 69, Linz, 4040 Austria

**Keywords:** Cementless THA; Femoral offset; Radiostereometric analysis; RSA, Migration

## Abstract

**Background:**

High-offset stems in cementless primary total hip arthroplasty (THA) have been potentially associated with early aseptic femoral loosening. This study aimed to evaluate the primary and secondary stability of a cementless high-offset femoral component under full weight-bearing conditions using model-based RSA, comparing it with a standard offset stem in patients undergoing THA.

**Methods:**

In this prospective, observational, single-center study, 42 patients with end-stage hip osteoarthritis underwent cementless primary THA using either a standard (SL-PLUS Standard) or a high-offset (SL-PLUS Lateral) cementless stem. Radiostereometric analysis (RSA) was employed to monitor stem migration at six weeks and three, six, twelve, and twenty-four months. Clinical outcomes were assessed using the modified Harris Hip Score (HHS) and the Western Ontario and McMaster Universities Osteoarthritis Index (WOMAC).

**Results:**

There were no significant differences in mean stem subsidence between the groups at any follow-up interval, indicating comparable primary and secondary stability. After minimal initial subsidence (SL-PLUS Standard: up to −0.54 mm; SL-PLUS Lateral: up to −0.73 mm), no further progressive migration was observed. A significant difference in stem anteversion was noted between the groups at six months (*P* = 0.021) and two years (*P* = 0.001). The SL-PLUS Lateral group had significantly better WOMAC scores at the two-year follow-up (*P* = 0.027).

**Conclusions:**

This RSA study demonstrated similar migration patterns for the high-offset and standard-offset cementless stems within the first two years after operation. Both groups exhibited initial subsidence followed by high secondary stability. Based on the results of this study, the SL-PLUS Lateral is a safe alternative for patients with high femoral offset undergoing cementless THA.

## Introduction

Total hip arthroplasty (THA) aims to restore native hip joint geometry and preoperative biomechanical conditions to optimize functional outcomes and implant survival [1–3]. An adequate reconstruction of the femoral offset (FO) correlates with improved abductor muscle function, increased hip stability, extended range of motion, and reduced polyethylene wear [1, 3–6]. However, the variability in native FOs often necessitates the use of lateralized femoral stems in patients with high offsets [7, 8]. A large FO can generally lead to increased strain at the implant-bone interface of the femoral component. Biomechanical studies suggested that high-offset stems lead to higher strains in the cement around cemented THA [9] by increasing the bending moment on the implant [10], increasing torsional loading along the long-axis [11], and contributing to increased micromotion in the upper stem zone [12]. In cementless THA, the resulting mechanical stress can potentially impede secondary osseointegration under full weight bearing, thereby promoting early aseptic loosening and implant failure [13–16]. However, other studies found no significant differences in micromotion between different offset versions when analyzed with 3-dimensional methods [17] and finite element models [18], but confirmed reduced risk of dislocation with high-offset stems, as each 1 mm offset increase allows a greater range of motion before impingement [19].

While registry studies have shown a detrimental effect of lateralized stem designs on implant fixation in cemented THA, with an associated risk of aseptic loosening, data regarding cementless high-offset stems are inconclusive. [16, 20, 21]. Recently published data have raised concerns about an increased risk of aseptic femoral loosening in patients treated with lateralized cementless stems [22, 23]. Jud et al. reported a 3.7-fold increased probability of aseptic loosening in a cohort of patients following cementless THA using a straight standard stem, when a high femoral offset combination was used [23].

The long-term stability of a hip implant after THA depends on primary fixation and subsequent osseointegration (secondary fixation). Initial fixation is established by the mechanical stability between the stem and bone. In the early postoperative period, loading can cause micromotions at the stem-bone interface. Micromotions can hinder osseointegration and compromise secondary fixation by promoting fibrous tissue formation [24–27]. Radiostereometric analysis (RSA) has demonstrated high accuracy and precision in detecting postoperative implant migration in vivo [28, 29]. Of note, increased stem migration in the first two years after surgery has been demonstrated to be a viable predictor for early implant failure [28–31]. However, to the best of our knowledge, no study has yet investigated the effects of increased FO on the primary and secondary stability of a cementless femoral component using RSA. This prospective controlled study aimed to assess the three-dimensional migration pattern of a cementless straight high-offset stem in vivo using model-based RSA and to investigate the influence of the FO on the primary and secondary stability of the implant under full weight bearing.

## Materials and methods

This prospective, observational, single-center study included a total of 42 patients with end-stage osteoarthritis of the hip who were indicated for primary THA. Based on native hip joint anatomy and preoperative digital planning using the TraumaCad software (Brainlab Inc., Westchester, USA), patients were allocated to receive either a standard (SL-PLUS Standard, Smith & Nephew Orthopaedics AG, Baar, Switzerland) or a high-offset cementless stem (SL-PLUS Lateral, Smith & Nephew Orthopaedics AG, Baar, Switzerland). The selection of the appropriate stem version aimed to reconstruct the hip joint geometry and restore the center of rotation. Both stem versions were digitally planned for each patient, and allocation was based on which stem design best replicated the patient’s native hip joint anatomy. Therefore, although the study included a control group (Standard stem), it was not randomized. We chose to compare different offset versions of the SL-Plus stem because its design is based on the Zweymüller stem, one of the most established and extensively studied stem designs. Its rectangular cross-sectional profile enables stable diaphyseal fixation, and the proximal hydroxyapatite (HA) coating promotes osseointegration.

Inclusion criteria were patients between 35 and 75 years of age with end-stage primary or secondary osteoarthritis of the hip and indication for cementless THA. Exclusion criteria were patients with rheumatoid arthritis, ongoing corticosteroid or osteoporosis treatment, hereditary skeletal diseases, or a history of corrective osteotomy of the proximal femur. Twenty patients were allocated to the high-offset group, and 22 to the control group. A sample size calculation was performed using the software G*Power (Version 3.1.9.3 for MacOS) [32]. The power analysis indicated that 17 patients per group would be needed to achieve a power of 80% in a two-sided *t*-test at a significance level of 0.05, assuming a clinically significant difference in stem migration between the two groups of 0.6 mm and a standard deviation of 0.6 mm based on previous study results [33, 34]. To account for potential dropouts and follow-up losses, at least 20 patients were recruited per group. The control group was also part of another clinical study conducted in parallel at our institution. Hence, the results of the SP-PLUS Standard group have already been published [35]. The study received approval from the local ethics committee (No. S-217/2007) and the Federal Office for Radiation Protection (No. Z 5–2246/2–2007-063) before the first patient was included. The study was conducted in accordance with the Helsinki Declaration, and written informed consent was obtained from all participants before study inclusion.

### Surgical technique

Two experienced senior surgeons (TG and SW) performed all surgical procedures according to the manufacturer’s surgical instructions. A modified anterolateral Bauer approach was used in all patients. Femoral reaming was standardized using a pneumatic broaching system (Woodpecker®, Integrated Medical Technologies USA, LLC, Minnesota, USA). Before implantation of the femoral stem, 5–10 radio-opaque tantalum markers with a diameter of 1.0 mm (Wennbergs Finmek AB, Gunnilse, Sweden), were implanted into the periprosthetic cancellous bone around the greater and lesser trochanter (Gruen zones 1 and 7) using the Halifax Bead Inserter (Halifax Biomedical Inc., Nova Scotia, Canada). Each patient received either the SL-PLUS Standard stem or the SL-PLUS Lateral stem (Smith & Nephew Orthopaedics AG, Baar, Switzerland) according to their native FO and based on the surgeon's intraoperative assessment. Both stems feature a straight, dual-tapered design with a rectangular cross-section and are made of grit-blasted titanium alloy (Ti6Al4Va) with a proximal hydroxyapatite (HA) coating. The proximal coating, which is supposed to facilitate bone ingrowth at the metaphyseal region, consists of a 0.3 mm open-pore titanium plasma layer and a 0.05 mm HA layer with a mean surface roughness of approximately 20–30 µm. The caput-collum-diaphyseal (CCD) angle of the SL-PLUS Standard stem is 131°, whereas the CCD angle of the SL-PLUS Lateral stem is 123° [36]. On the acetabular side, a cementless press-fit titanium shell was used in combination with a highly crosslinked polyethylene inlay (Allofit® cup and Durasul® insert, Zimmer Biomet, Warsaw, IN, USA). Except for one patient who required a cobalt-chromium alloy metal head due to a large head length, all patients received a 32 mm ceramic femoral head (BIOLOX® forte, CeramTec GmbH, Plochingen, Germany). Patients were mobilized under full weight bearing immediately after surgery.

### Radiostereometric analysis (RSA)

**Fig. 1:**
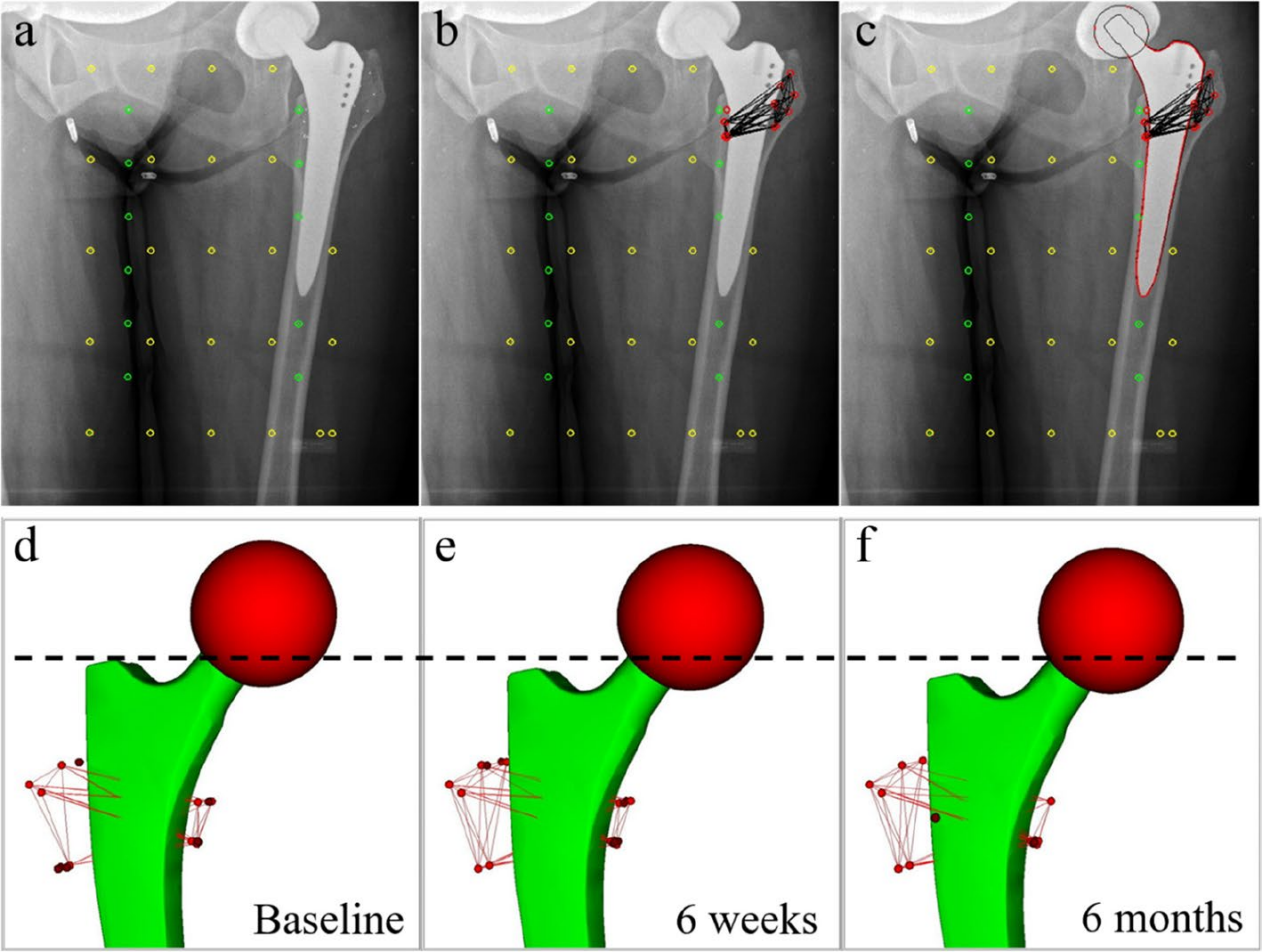
Model-based RSA process for tracking stem migration over time. **a** AP radiograph showing reference markers (yellow) and control markers (green) used for calibration. **b** Visualization of the tantalum beads (red), inserted during surgery into the cancellous bone as femoral markers. **c** Implant outline serves as the basis for the 3D surface model of the implant, with its actual projection relative to the markers, used for assessing the implant’s position and rotation by matching the two synchronized radiographic stereo images. **d**–**f** Illustrations of a 3D implant model visualizing stem migration from (**d**) baseline, through (**e**) 6 weeks, to (**f**) 6 months post-THA, with the dashed line indicating the level of the stem shoulder at baseline

The primary objective of this study was to evaluate and compare the primary and secondary stability of the femoral component in both the study and the control group by monitoring stem migration during the first two years after surgery using the model-based RSA technique. To obtain unipolar stereo images, two synchronized X-ray tubes were set at a 40-degree angle to each other. A carbon filter calibration box (Medis Specials, Leiden, Netherlands) was placed under the patient’s joint, with two digital film cassettes positioned in the lower plane of the box. The X-ray exposure settings were standardized at 90 kV and 12.5 mAs across all images. For image analysis, the model-based RSA software (Version 3.3, Medis Specials, Leiden, Netherlands) was used. Adhering to the RSA guidelines, the mean error for rigid body fitting was limited to a maximum of 0.35 mm, and the condition number was restricted to 150 to ensure adequate marker stability and distribution [37]. At each follow-up interval, the linear migration of the stem relative to the baseline was measured in terms of rotation and translation along all three axes. RSA assessments were conducted one-week post-surgery (as the baseline), and subsequently at 6 weeks, 3 months, 6 months, 12 months, and 24 months postoperatively (Fig. 1).

To verify the precision of the RSA system, double examinations were performed on 28 patients at the 6-month follow-up visit. As part of the double examination, the patients were repositioned between the RSA measurements, and the mean value and the standard deviation (SD) of the difference between the two measurements were calculated for all patients [37, 38]. The precision level was then calculated using the following formula, where $P$ represents precision, $x$ is the difference between the double examinations, and 2.048 represents the critical value in a 95% t-distribution for a sample size of *n* = 28 [39].

$$P=2.048\times SD=2.048\times \sqrt{\frac{\sum_{i=1}^{n}{({x}_{i})}^{2}}{n}}$$

### Clinical and radiological evaluation

The secondary objective of this study was to evaluate differences in clinical results between the two groups using the modified Harris Hip Score (HHS) and the Western Ontario and McMaster Universities Osteoarthritis Index (WOMAC) as outcome measures [40]. The HHS ranges from 0 (worst) to 100 (best) and includes assessments of pain, function and range of motion [40]. The WOMAC ranges from 96 (worst) to 0 (best) and is a patient-reported outcome measure, covering the sub-items pain, stiffness, and functional limitations [41]. Clinical outcomes were scored preoperatively and at the 3-month, 12-month, and 24-month follow-up visits. In addition, standard anteroposterior radiographs of the lateral radiographs the hip were taken for radiological evaluation pre- and postoperatively, and subsequently at 3, 12, and 24 months. Radiographs were analyzed for the presence of radiolucent lines (>2 mm), signs of cortical hypertrophy and the presence of heterotopic ossifications (HO), which were graded according to the Brooker classification system [42].

### Statistical analysis

Data were evaluated descriptively as arithmetic mean, standard deviation (SD), minimum and maximum values. The Shapiro–Wilk test demonstrated that the data were normally distributed. To compare demographic data and differences in stem migration between the two groups at a given time point, the Student’s *t*-test for independent samples was used. For comparing differences in stem migration across different time intervals within each group, analysis of variance for repeated measures (ANOVA) with Tukey’s test for multiple comparisons was applied. The chi-square test and Fisher’s exact test were used to compare categorical variables between the two groups. All tests were two-sided, and a *P*-value < 0.05 was considered statistically significant. Statistical analysis was performed using the software SPSS® for Windows® (version 29.0; SPSS IBM Corp., Chicago, IL, USA) and Graphpad Prism® (version 10.0, Graphpad Software, San Diego, CA, USA).

## Results

**Fig. 2:**
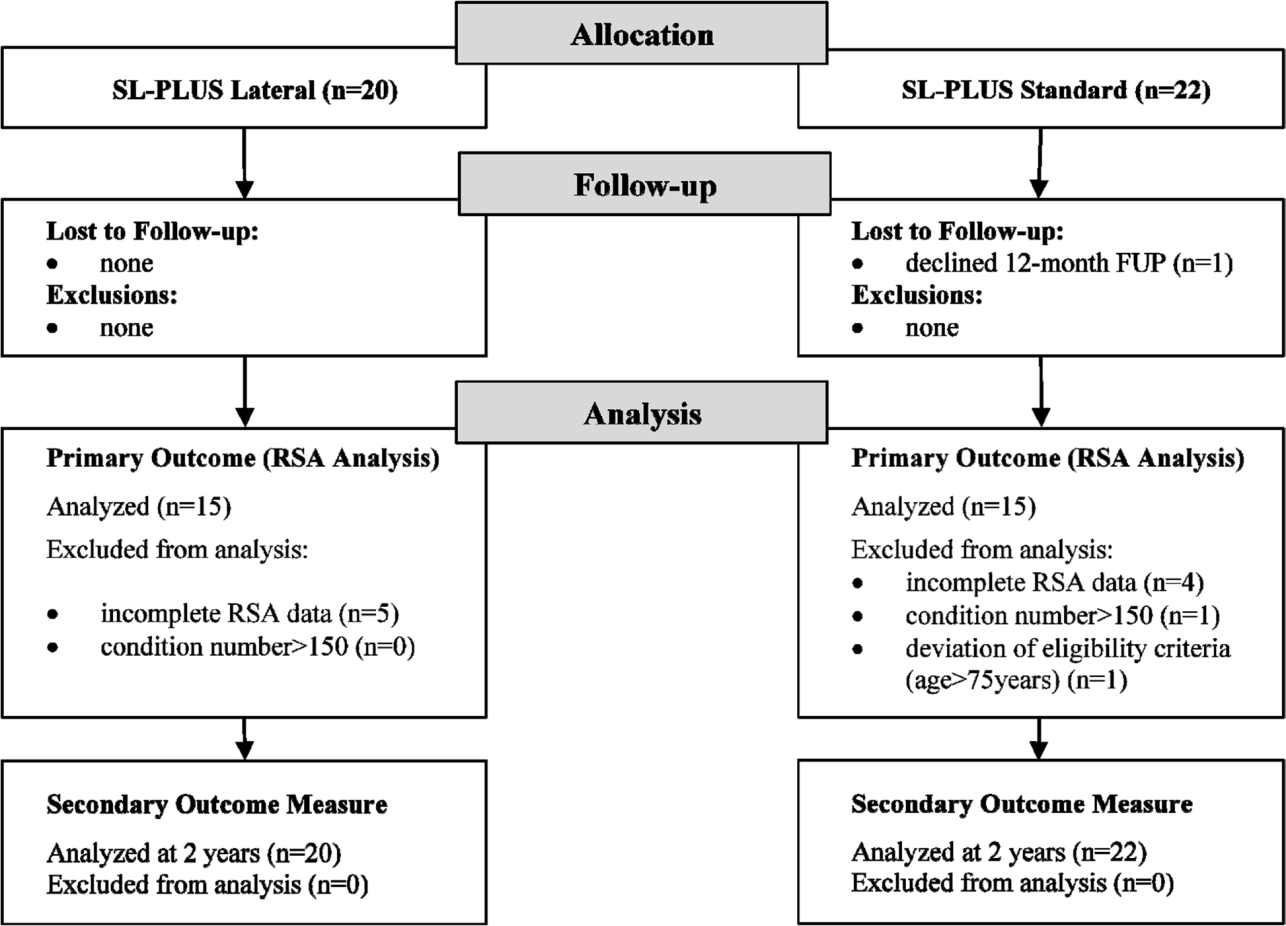
Study flowchart demonstrating the follow-up and analysis of both study cohorts

Demographic data of the study and control groups are summarized in Table 1. Except for the distribution of genders, there were no statistically significant differences in the demographic variables between the two groups. One patient was lost to follow-up because he refused the 12-month follow-up visit. A total of nine patients with insufficient marker detection and one patient with a condition number > 150 were excluded from the RSA analysis. Another patient, who did not meet the eligibility criteria, was excluded from the control group. This left a total of 15 patients in the SL-PLUS Lateral group and 15 patients in the SL-PLUS Standard group with complete RSA data sets available for final RSA analysis. Figure 2 illustrates the study flow and follow-up of patients.

**Table 1:** Demographic data of the study group (SL-PLUS Lateral) and the control group (SL-PLUS Standard)

Parameter	SL-PLUS Lateral (*n* = 20)	SL-PLUS Standard (*n* = 22)	*P*-value
Age at surgery^a^ (years)	58.6 (SD 11.2)	60.7 (SD 10.1)	0.51
Gender (female/male) (*n*)	5/15	14/8	**0.01**
Operated hip (right/left) (*n*)	9/11	12/10	0.54
BMI^a^ (kg/m^2^)	28.8 (SD 5.6)	26.5 (SD 4.7)	0.16
Head length (S/M/L/XL) (*n*)	5/11/4/0	7/6/8/1	0.27
HHS preoperatively^a^ (points)	51.2 (SD 11.2)	48.8 (SD 14.8)	0.56
WOMAC preop.^a^ (points)	52.4 (SD 18.8)	56.7 (SD 17.1)	0.45

^a^The values are given as the mean, with the SD in parentheses

*BMI* Body mass index, *HHS* Harris Hip Score, *WOMAC* Western Ontario and McMaster Universities Osteoarthritis Index

One complication was reported in the control group at the 3-month follow-up visit with a trochanteric tip fracture in a 57-year-old male patient, which did not require revision surgery or further treatment. The patient was pain-free and reported to be satisfied with the outcome of the hip replacement.

To prove the reproducibility of the RSA method, we performed a precision analysis through double examination measurements 6 months after the surgical procedure. We observed RSA precision levels for translational measurements of 0.12 mm, 0.23 mm, and 0.63 mm and rotational measurements of 0.64°, 1.33°, and 0.35° along the frontal, longitudinal, and sagittal axis, respectively.

The results of the RSA analysis demonstrated no statistically significant differences in mean stem subsidence (translational migration along the stem axis) between the two groups at any follow-up (Table 2). Mean stem subsidence ranged from −0.54 to −0.73 mm for the SL-PLUS Lateral stem and from −0.40 to −0.54 mm for the SL-PLUS Standard stem.

**Table 2:** RSA measurement results of femoral stem migration and rotation

		**SL-PLUS Lateral (*n* = 15)**		**SL-PLUS Standard (*n* = 15)**		**Lateral vs. Standard**
	**Interval**	**Mean (SD)**	**(95% CI)**	**Mean (SD)**	**(95% CI)**	***P*-value**
**Translation (mm)**						
Proximal( +)/Distal( −)	6 weeks	−0.73 (0.72)	−1.12 to −0.33	−0.41 (0.83)	−0.86 to 0.05	0.266
	3 months	−0.59 (0.67)	−0.96 to −0.22	−0.48 (0.64)	−0.84 to −0.13	0.649
	6 months	−0.59 (0.63)	−0.94 to −0.24	−0.54 (0.67)	−0.90 to −0.17	0.829
	12 months	−0.58 (0.68)	−0.96 to −0.21	−0.45 (0.76)	− 0.87 to −0.02	0.604
	24 months	−0.54 (0.65)	−0.90 to −0.18	−0.40 (0.66)	−0.77 to −0.04	0.578
**Rotation (degrees)**						
Ante-( −)/Retroversion( +)	6 weeks	0.37 (1.82)	−0.63 to 1.38	−0.12 (1.95)	−1.19 to 0.96	0.484
	3 months	0.02 (2.45)	−1.34 to 1.38	−0.48 (1.76)	−1.46 to 0.49	0.523
	6 months	0.72 (2.48)	−0.65 to 2.10	−1.15 (1.64)	−2.06 to −0.25	0.021*
	12 months	0.33 (2.18)	−0.87 to 1.54	−0.66 (1.48)	−1.48 to 0.16	0.155
	24 months	1.25 (1.95)	0.17 to 2.33	−1.29 (1.81)	−2.30 to −0.29	0.001*
**Rotation (degrees)**						
Valgus-( +)/Varus( −), Tilt	6 weeks	−0.29 (0.95)	−0.81 to 0.24	0.02 (0.19)	−0.09 to 0.13	0.231
	3 months	−0.01 (0.52)	−0.30 to 0.27	0.07 (0.51)	−0.21 to 0.36	0.651
	6 months	−0.22 (0.99)	−0.77 to 0.33	0.09 (0.47)	−0.18 to 0.35	0.288
	12 months	−0.11 (0.85)	−0.57 to 0.36	0.01 (0.41)	−0.22 to 0.24	0.633
	24 months	−0.13 (0.68)	−0.51 to 0.25	0.04 (0.39)	−0.18 to 0.25	0.412
*P < 0.05						

**Fig. 3:**
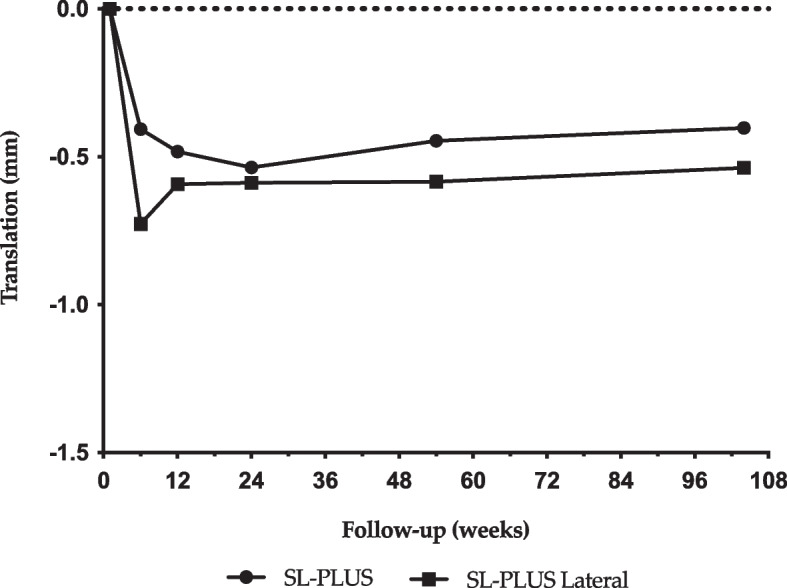
Mean stem subsidence for SL-PLUS and SL-PLUS Lateral stem designs over time

The SL-PLUS Lateral stem showed statistically significant subsidence during the first six weeks postoperatively (ANOVA, *P* = 0.015, Table 3), indicating initial settling of the stem under full weight bearing (Fig. 3). Both stem designs exhibited high secondary stability and optimal osseointegration after initial settling without signs of persistent migration up to the latest follow-up. A statistically significant difference in stem anteversion was seen between the two groups after six months (*P* = 0.021) and two years (*P* = 0.001, Table 2). However, the accuracy of the RSA method for detecting changes in stem rotation along the longitudinal axis should be considered a limitation when interpreting these results. No other statistically significant differences in stem rotation along the longitudinal and sagittal axis or stem translation in these planes were observed between the two groups or over the follow-up intervals.

**Table 3:** Comparison of stem subsidence between adjacent follow-up intervals within each group

Group	Follow-up interval	Mean of difference	95% CI of difference	*P*-value
**SL-PLUS Lateral**	Baseline vs. 6 weeks	0.73	0.12 to 1.33	0.015*
	6 weeks vs. 3 months	− 0.13	− 0.44 to 0.17	0.712
	3 months vs. 6 months	− 0.01	− 0.26 to 0.25	> 0.999
	6 months vs. 1 year	− 0.003	− 0.16 to 0.16	> 0.999
	1 year vs. 2 years	− 0.05	− 0.21 to 0.12	0.927
**SL-PLUS Standard**	Baseline vs. 6 weeks	0.41	− 0.29 to 1.11	0.439
	6 weeks vs. 3 months	0.08	− 0.20 to 0.36	0.942
	3 months vs. 6 months	0.05	− 0.07 to 0.18	0.739
	6 months vs. 1 year	− 0.09	− 0.34 to 0.15	0.824
	1 year vs. 2 years	− 0.04	− 0.27 to 0.18	0.987
*P < 0.05

This table presents the results of RSA measurements for femoral stem migration (translation), stem rotation along the longitudinal axis (ante-/retroversion), and stem rotation along the sagittal axis (varus/valgus tilt) for both SL-PLUS Lateral and SL-PLUS Standard stem designs at each follow-up interval. The data includes the mean values, standard deviations (SD), 95% confidence intervals (CI), and *p*-values comparing the two groups with * indicating statistically significant differences between the study and the control group.

This table compares stem subsidence at different follow-up intervals within the SL-PLUS Lateral and SL-PLUS Standard groups. The data includes the mean difference in subsidence, 95% confidence intervals (CI) for the difference, and *p*-values for each comparison, with^.*^ indicating statistically significant differences.

This figure shows the mean stem subsidence of the SL-PLUS (circles) and SL-PLUS Lateral (square) stem designs, measured over a period of up to two years post-surgery. The x-axis represents the follow-up time in weeks, while the y-axis indicates the amount of stem subsidence in millimeters (translation). Data points illustrate the mean subsidence for each design at different time points.

Conventional radiographs at the two-year follow-up demonstrated no signs of loosening or progressive radiolucent lines. Seven patients (35%) in the SL-PLUS Lateral group and 7 patients (32%) in the SL-PLUS Standard group exhibited heterotopic ossifications (HO) Brooker grade I or II. No patient had HO grade III or IV. Femoral cortical hypertrophy was more frequently seen in the SL-PLUS Lateral group (*n* = 4; 20%) when compared to the control group (*n* = 1; 5%), although the difference was not statistically significant (*P* = 0.12).

There was a significant postoperative improvement in mean HHS and WOMAC at the 2-year interval compared to the baseline (*P* < 0.0001). At two year follow-up, the mean HHS was 94 points (SD 9.2) in the SL-PLUS Lateral group and 89 points (SD 11.9) in the SL-PLUS Standard group. The mean WOMAC score was 2.1 points (SD 3.7) in the SL-PLUS Lateral group and 7.3 points (SD 8.9) in the SL-PLUS Standard group. The difference in WOMAC scores between the two groups was statistically significant (*P* = 0.03).

## Discussion

This study aimed to assess the primary and secondary stability of a cementless high-offset femoral component in vivo under full weight bearing using the model-based RSA technique and to compare implant stability with a control group of patients, who underwent cementless THA with a standard offset stem. The results of our study demonstrated that an increased femoral offset did not adversely affect the primary and secondary stability of the femoral component. Both stem designs exhibited high primary stability, with only minimal subsidence (< 1 mm) observed during the initial postoperative weeks. Additionally, they showed excellent secondary stability, characterized by good osseointegration and an absence of progressive implant migration up to the final follow-up.

Adequate reconstruction of the native FO is crucial for achieving satisfactory functional outcomes, optimal hip stability, and muscle strength by enhancing the lever arm for abduction [1–3]. However, the increased lever arm also leads to greater strain on the femoral component, particularly in the varus direction and retroversion. This added strain can cause excessive implant migration and early aseptic loosening [13, 43]. In cementless THA, these increased mechanical stresses can potentially adversely affect both primary stabilities, leading to excessive implant migration in the first postoperative weeks, and secondary stability due to increased micromotions. These micromotions can compromise secondary osseointegration and potentially lead to early implant failure due to aseptic loosening [13–16]. Indeed, micromotions exceeding 150 μm have been shown to hinder osseointegration and compromise secondary fixation by promoting the formation of fibrous tissue [24–27].

Registry data have demonstrated an increased failure risk for high offset stems in cemented THA [15, 16, 43]. A study from the Norwegian Arthroplasty Register reported a 3.3 times higher relative risk for stem revision when a lateralized stem was used [43]. Similarly, another study from the Swedish Hip Arthroplasty Register, which analyzed 71,184 primary THA, found a comparable increase in revision risk for cemented high-offset stems [15]. However, the published data regarding the revision risk of high-offset stems in cementless THA are inconclusive. Jameson et al. investigated independent predictors of failure in 35,386 cementless single-brand total hip replacements (Corail/Pinnacle, DePuy Synthes) using data from the National Joint Registry for England and Wales and found no correlation between stem offset and the risk of revision [20]. In contrast, Melbye et al. analyzed the survivorship of different cementless Corail stem variants in 51,212 THA from the Norwegian Arthroplasty Register and reported an increased revision risk for aseptic loosening associated with the high-offset stems compared to the standard stems [21]. Similarly, Cantin et al. compared the survival rates of 807 primary THA using the cementless Corail stem after a mean follow-up of 2.3 years and found an increased risk for aseptic loosening associated with the high-offset stem version [22]. This finding is consistent with a recently published study by Jud et al., who reported a 3.7-fold increased risk of aseptic femoral loosening in a cohort of 2,459 cementless THA when a high femoral offset combination was used [23]. The authors concluded that an adjustment of the postoperative protocol might be necessary for these patients to ensure adequate stem ingrowth [23]. Notably, the inclusion of various stem designs in these registry studies may contribute to the conflicting results observed across different studies.

To our knowledge, no study has yet investigated the migration pattern of a cementless high-offset stem in vivo under full-weight bearing using RSA. Model-based RSA is highly accurate in detecting stem migration in vivo and has proven to be a viable and reliable method for the early detection of potential implant failures [44]. The stem subsidence measured in our study was minimal for both stem designs and occurred primarily during the first six weeks postoperatively, representing initial settling. After this period, no implant demonstrated excessive migration beyond the critical threshold of 0.5 to 1.0 mm, which is considered to be associated with an increased risk of clinical failure and aseptic loosening [45]. Both stem designs share key structural features, such as a rectangular cross-sectional profile, which likely contributes to stable diaphyseal fixation, irrespective of the offset version. Additionally, the proximal hydroxyapatite (HA) coating on both stems enhances osseointegration, promoting secondary stability and preventing further migration after the initial settling period. Our findings are consistent with the results of Fottner and colleagues [17], who found no significant differences in micromotions between cementless hip prostheses with varying offsets under physiological loading conditions. The use of the same pneumatic broaching system for femoral reaming in both groups likely standardized the surgical procedure, minimizing variability in stem fixation. Early postoperative full weight-bearing may have further contributed to the comparable outcomes by stabilizing both stem designs similarly.

Although there was a statistically significant difference in mean stem rotation along the longitudinal axis (ante-/retroversion) between the high-offset and the control groups at the six-month and two-year follow-up, no statistically significant changes in stem rotation over time were observed within each group. It is important to consider the limited precision of the RSA method for detecting rotational changes along the longitudinal axis, which may be a limitation in interpreting these results [46]. Based on recent RSA studies [47], mean rotational changes of up to 2.4° have been reported without clinical impact. In our study, the differences in ante-/retroversion at 6 and 24 months, though statistically significant, likely fall within the RSA method’s precision limits, as indicated by our precision analysis (double examination measurements) showing a level of 1.33° for rotational measurements along the longitudinal axis. Thus, these differences are most likely due to the accuracy limitations of RSA rather than prosthesis design, surgical technique, or patient-related factors.

The stem migration rates measured in our study and the precision levels assessed by double examination at the six-month follow-up interval, align closely with the results of other RSA studies that have been reported in the literature [33, 39]. Nysted et al. reported an RSA precision level of 0.21 mm for translation and 1.36 degrees for rotation around the longitudinal axis, which corresponds well with the precision measured in our study [39]. The standard deviations for subsidence in our study, ranging from 0.63 to 0.83 mm across both stem types, were also within the normal range reported in RSA studies. Given the precision level of 0.23 mm for translational measurements in our study, this variability is not unexpected. Additionally, another study by Hoornenborg et al. investigated the migration pattern of the same femoral component (SL-PLUS Standard) using RSA [33]. They reported a mean stem subsidence of 0.46 mm (range −2.17 to 0.05 mm) after two years, which is consistent with the results of our study [33]. We acknowledge that a further subgroup analysis based on patient factors, such as BMI or gender, could help explain individual variations in migration. However, a larger sample size would be required for a more detailed analysis, and we plan to address this in future studies.

Some limitations of this study should be acknowledged. We noticed a high rate of incomplete RSA data in our cohort, leading to the exclusion of 9 patients from the final RSA migration analysis. This issue was primarily due to insufficient marker detection, which is the main limitation of this study, as it influenced the study's statistical power. It highlights the importance of an adequate positioning of the tantalum beads during surgery to ensure sufficient marker detection. This consideration should be prioritized in future RSA studies. A wide distribution of the tantalum markers around the prosthesis is beneficial for enhancing the accuracy and precision of RSA measurements. Another limitation is the unequal gender distribution between the groups, with a predominance of male subjects (75%) in the SL-PLUS lateral group compared to the control group (36%). This discrepancy can be attributed to gender-related anatomical differences in native hip joint geometry, as a higher prevalence of large FOs in male subjects often necessitates the use of a high-offset stem [21]. Previous studies have identified male sex as an independent risk factor for stem revision [21, 43]. In fact, male sex was associated with a relative risk (RR) of revision that was 2.5 times higher than that for females, specifically for stem revision due to aseptic stem loosening [43]. Gender differences in hip joint anatomy (e.g., offset, leg length, osteoporosis) must therefore be recognized during reconstructive hip surgery with THA [48]. Our study did not confirm this finding. However, we recognize that this gender imbalance may introduce bias and limit the generalizability of the results. To mitigate this limitation in future studies, we suggest stratifying patients by gender during the study design phase (e.g., age- and gender-matched randomization) to ensure a more balanced distribution. Additionally, increasing the sample size may help account for any gender-related variability.

Additionally, the single-center study design may limit the generalizability of our results. Although the controlled environment allowed for standardized surgical procedures and postoperative care, a multicenter randomized follow-up study could increase the level of evidence. Finally, while the procedures were performed by two experienced surgeons using the same surgical approach and pneumatic system for femoral reaming, we recognize that surgeon expertise may affect the reproducibility of these outcomes in other settings with varying levels of experience.

## Conclusions

In conclusion, our RSA study demonstrated similar migration patterns for the high-offset version of a cementless dual-tapered straight femoral stem compared to the standard stem within the first two years after surgery. Both groups exhibited initial subsidence under full weight bearing, followed by high secondary stability and good osseointegration. Although the sample size was relatively small, with a high proportion of missing RSA data, our findings provided valuable insights. A larger cohort with equal gender distribution would further strengthen the conclusions. Based on the clinical results of our study, the SL-PLUS Lateral is a safe alternative for patients with high FO undergoing cementless THA and a postoperative rehabilitation protocol with mobilization under full weight bearing is feasible for these patients to ensure adequate osseointegration.

## Data Availability

The datasets used and/or analyzed during the current study are available from the corresponding author on reasonable request.

## Abbreviations

ANOVA: Analysis of variance
BMI: Body Mass Index
CCD: Caput-collum-diaphyseal
FO: Femoral offset
HA: Hydroxyapatite
HHS: Harris Hip Score
RSA: Radiostereometric analysis
SD: Standard deviation
THA: Total hip arthroplasty
WOMAC: Western Ontario and McMaster Universities Osteoarthritis Index

## References

[1] Charles MN, Bourne RB, Davey JR, Greenwald AS, Morrey BF, Rorabeck CH. Soft-tissue balancing of the hip: the role of femoral offset restoration. Instr Course Lect. 2005;54:131–41. 15948440

[2] Mahmood SS, Mukka SS, Crnalic S, Wretenberg P, Sayed-Noor AS. Association between changes in global femoral offset after total hip arthroplasty and function, quality of life, and abductor muscle strength. A prospective cohort study of 222 patients. Acta Orthop. 2016;87(1):36–41. 26471772 10.3109/17453674.2015.1091955 PMC4940589

[3] McGrory BJ, Morrey BF, Cahalan TD, An KN, Cabanela ME. Effect of femoral offset on range of motion and abductor muscle strength after total hip arthroplasty. J Bone Joint Surg Br. 1995;77(6):865–9. 7593096

[4] Devane PA, Horne JG. Assessment of polyethylene wear in total hip replacement. Clin Orthop Relat Res. 1999;369:59–72. 10.1097/00003086-199912000-00007 10611861

[5] Renkawitz T, Weber T, Dullien S, Woerner M, Dendorfer S, Grifka J, et al. Leg length and offset differences above 5mm after total hip arthroplasty are associated with altered gait kinematics. Gait Posture. 2016;49:196–201. 27450670 10.1016/j.gaitpost.2016.07.011

[6] Weber M, Merle C, Nawabi DH, Dendorfer S, Grifka J, Renkawitz T. Inaccurate offset restoration in total hip arthroplasty results in reduced range of motion. Sci Rep. 2020;10(1):13208. 32764592 10.1038/s41598-020-70059-1 PMC7413373

[7] Bachour F, Marchetti E, Bocquet D, Vasseur L, Migaud H, Girard J. Radiographic preoperative templating of extra-offset cemented THA implants: how reliable is it and how does it affect survival? Orthop Traumatol Surg Res. 2010;96(7):760–8. 20851077 10.1016/j.otsr.2010.05.004

[8] Rubin PJ, Leyvraz PF, Aubaniac JM, Argenson JN, Esteve P, de Roguin B. The morphology of the proximal femur. A three-dimensional radiographic analysis. J Bone Joint Surg Br. 1992;74(1):28–32. 1732260 10.1302/0301-620X.74B1.1732260

[9] Kleemann RU, Heller MO, Stoeckle U, Taylor WR, Duda GN. THA loading arising from increased femoral anteversion and offset may lead to critical cement stresses. J Orthop Res. 2003;21(5):767–74. 12919861 10.1016/S0736-0266(03)00040-8

[10] Davey JR, O’Connor DO, Burke DW, Harris WH. Femoral component offset. Its effect on strain in bone-cement. J Arthroplasty. 1993;8(1):23–6. 8436985

[11] Peng L, Ma J, Zeng Y, Wu Y, Si H, Shen B. Clinical and radiological results of high offset tri-lock bone preservation stem in unilateral primary total hip arthroplasty at a minimum follow-up of 3 years. J Orthop Surg Res. 2021;16(1):635. 34689823 10.1186/s13018-021-02787-7 PMC8543806

[12] Meisterhans M, Dimitriou D, Fasser M-R, Hoch A, Jud L, Zingg PO. Influence of offset on osseointegration in cementless total hip arthroplasty: a finite element study. J Orthop Res. 2024;42(7):1566–76. 38376065 10.1002/jor.25808

[13] Asayama I, Chamnongkich S, Simpson KJ, Kinsey TL, Mahoney OM. Reconstructed hip joint position and abductor muscle strength after total hip arthroplasty. J Arthroplasty. 2005;20(4):414–20. 16124955 10.1016/j.arth.2004.01.016

[14] Ramaniraka NA, Rakotomanana LR, Rubin PJ, Leyvraz P. Noncemented total hip arthroplasty: influence of extramedullary parameters on initial implant stability and on bone-implant interface stresses. Rev Chir Orthop Reparatrice Appar Mot. 2000;86(6):590–7. 11060433

[15] Thien TM, Karrholm J. Design-related risk factors for revision of primary cemented stems. Acta Orthop. 2010;81(4):407–12. 20586706 10.3109/17453674.2010.501739 PMC2917561

[16] Wyatt MC, Kieser DC, Kemp MA, McHugh G, Frampton CMA, Hooper GJ. Does the femoral offset affect replacements? The results from a National Joint Registry. Hip Int. 2019;29(3):289–98. 29873253 10.1177/1120700018780318

[17] Fottner A, Peter CV, Schmidutz F, Wanke-Jellinek L, Schröder C, Mazoochian F, et al. Biomechanical evaluation of different offset versions of a cementless hip prosthesis by 3-dimensional measurement of micromotions. Clin Biomech (Bristol, Avon). 2011;26(8):830–5. 21536357 10.1016/j.clinbiomech.2011.04.001

[18] Simpson DJ, Little JP, Gray H, Murray DW, Gill HS. Effect of modular neck variation on bone and cement mantle mechanics around a total hip arthroplasty stem. Clin Biomech (Bristol, Avon). 2009;24(3):274–85. 19263573 10.1016/j.clinbiomech.2008.12.010

[19] Vigdorchik JM, Sharma AK, Elbuluk AM, Carroll KM, Mayman DJ, Lieberman JR. High Offset Stems Are Protective of Dislocation in High-Risk Total Hip Arthroplasty. J Arthroplasty. 2021;36(1):210–6. 32741711 10.1016/j.arth.2020.07.016

[20] Jameson SS, Baker PN, Mason J, Rymaszewska M, Gregg PJ, Deehan DJ, et al. Independent predictors of failure up to 7.5 years after 35 386 single-brand cementless total hip replacements: a retrospective cohort study using National Joint Registry data. Bone Joint J. 2013;95-B(6):747–57. 23723267 10.1302/0301-620X.95B6.31378

[21] Melbye SM, Haug SCD, Fenstad AM, Furnes O, Gjertsen JE, Hallan G. How does implant survivorship vary with different corail femoral stem variants? Results of 51,212 cases with up to 30 years of follow-up from the Norwegian Arthroplasty Register. Clin Orthop Relat Res. 2021;479(10):2169–80. 34427568 10.1097/CORR.0000000000001940 PMC8445552

[22] Cantin O, Viste A, Desmarchelier R, Besse JL, Fessy MH. Compared fixation and survival of 280 lateralised vs 527 standard cementless stems after two years (1–7). Orthop Traumatol Surg Res. 2015;101(7):775–80. 26476972 10.1016/j.otsr.2015.08.002

[23] Jud L, Ruedi N, Dimitriou D, Hoch A, Zingg PO. High femoral offset as a risk factor for aseptic femoral component loosening in cementless primary total hip arthroplasty. Int Orthop. 2024;48(5):1217–24. 38388804 10.1007/s00264-024-06116-5 PMC11001651

[24] Pilliar RM, Lee JM, Maniatopoulos C. Observations on the effect of movement on bone ingrowth into porous-surfaced implants. Clin Orthop Relat Res. 1986;208:108–13. 3720113

[25] Jasty M, Bragdon C, Burke D, O’Connor D, Lowenstein J, Harris WH. In vivo skeletal responses to porous-surfaced implants subjected to small induced motions. J Bone Joint Surg Am. 1997;79(5):707–14. 9160943 10.2106/00004623-199705000-00010

[26] Engh CA, O’Connor D, Jasty M, McGovern TF, Bobyn JD, Harris WH. Quantification of implant micromotion, strain shielding, and bone resorption with porous-coated anatomic medullary locking femoral prostheses. Clin Orthop Relat Res. 1992;285:13–29. 1446429

[27] Watanabe R, Mishima H, Totsuka S, Nishino T, Yamazaki M. Primary stability of collared and collarless cementless femoral stems - a finite element analysis study. Arthroplast Today. 2023;21:101140. 37151402 10.1016/j.artd.2023.101140 PMC10160691

[28] Hurschler C, Seehaus F, Emmerich J, Kaptein BL, Windhagen H. Accuracy of model-based RSA contour reduction in a typical clinical application. Clin Orthop Relat Res. 2008;466(8):1978–86. 18509712 10.1007/s11999-008-0287-3 PMC2584241

[29] Nieuwenhuijse MJ, Valstar ER, Kaptein BL, Nelissen RG. Good diagnostic performance of early migration as a predictor of late aseptic loosening of acetabular cups: results from ten years of follow-up with Roentgen stereophotogrammetric analysis (RSA). J Bone Joint Surg Am. 2012;94(10):874–80. 22617914 10.2106/JBJS.K.00305

[30] Valstar E, Kaptein B, Nelissen R. Radiostereometry and new prostheses. Acta Orthop. 2012;83(2):103–4. 22489889 10.3109/17453674.2012.678796 PMC3339520

[31] van der Voort P, Pijls BG, Nieuwenhuijse MJ, Jasper J, Fiocco M, Plevier JW, et al. Early subsidence of shape-closed hip arthroplasty stems is associated with late revision. A systematic review and meta-analysis of 24 RSA studies and 56 survival studies. Acta Orthop. 2015;86(5):575–85. 25909455 10.3109/17453674.2015.1043832 PMC4564780

[32] Faul F, Erdfelder E, Buchner A, Lang AG. Statistical power analyses using G*Power 3.1: tests for correlation and regression analyses. Behav Res Methods. 2009;41(4):1149–60. 19897823 10.3758/BRM.41.4.1149

[33] Hoornenborg D, Sierevelt IN, Spuijbroek JA, Cheung J, van der Vis HM, Beimers L, et al. Does hydroxyapatite coating enhance ingrowth and improve longevity of a Zweymuller type stem? A double-blinded randomised RSA trial. Hip Int. 2018;28(2):115–21. 28967054 10.5301/hipint.5000549

[34] Onsten I, Carlsson AS, Sanzen L, Besjakov J. Migration and wear of a hydroxyapatite-coated hip prosthesis. A controlled roentgen stereophotogrammetric study. J Bone Joint Surg Br. 1996;78(1):85–91. 8898134

[35] Reiner T, Sonntag R, Kretzer JP, Clarius M, Jakubowitz E, Weiss S, et al. The migration pattern of a cementless hydroxyapatite-coated titanium stem under immediate full weight-bearing-a randomized controlled trial using model-based RSA. J Clin Med. 2020;9(7):2077. 32630629 10.3390/jcm9072077 PMC7408977

[36] Smith and Nephew Orthopaedics: AG. https://www.smith-nephew.com/en/health-care-professionals/products/orthopaedics/sl-plus-mia#surgicaltechniques.

[37] Valstar ER, Gill R, Ryd L, Flivik G, Borlin N, Karrholm J. Guidelines for standardization of radiostereometry (RSA) of implants. Acta Orthop. 2005;76(4):563–72. 16195075 10.1080/17453670510041574

[38] Ranstam J, Ryd L, Onsten I. Accurate accuracy assessment: review of basic principles. Acta Orthop Scand. 2000;71(1):106–8. 10744004 10.1080/00016470052944017

[39] Nysted M, Foss OA, Klaksvik J, Benum P, Haugan K, Husby OS, et al. Small and similar amounts of micromotion in an anatomical stem and a customized cementless femoral stem in regular-shaped femurs. A 5-year follow-up randomized RSA study. Acta Orthop. 2014;85(2):152–8. 24650024 10.3109/17453674.2014.899846 PMC3967257

[40] Mahomed NN, Arndt DC, McGrory BJ, Harris WH. The Harris hip score: comparison of patient self-report with surgeon assessment. J Arthroplasty. 2001;16(5):575–80. 11503116 10.1054/arth.2001.23716

[41] Bellamy N, Buchanan WW, Goldsmith CH, Campbell J, Stitt LW. Validation study of WOMAC: a health status instrument for measuring clinically important patient relevant outcomes to antirheumatic drug therapy in patients with osteoarthritis of the hip or knee. J Rheumatol. 1988;15(12):1833–40. 3068365

[42] Brooker AF, Bowerman JW, Robinson RA, Riley LH Jr. Ectopic ossification following total hip replacement. Incidence and a method of classification. J Bone Joint Surg Am. 1973;55(8):1629–32. 4217797

[43] Hallan G, Espehaug B, Furnes O, Wangen H, Hol PJ, Ellison P, et al. Is there still a place for the cemented titanium femoral stem? 10,108 cases from the Norwegian Arthroplasty Register. Acta Orthop. 2012;83(1):1–6. 22206445 10.3109/17453674.2011.645194 PMC3278649

[44] Nelissen RG, Pijls BG, Karrholm J, Malchau H, Nieuwenhuijse MJ, Valstar ER. RSA and registries: the quest for phased introduction of new implants. J Bone Joint Surg Am. 2011;93(Suppl 3):62–5. 22262426 10.2106/JBJS.K.00907

[45] Karrholm J, Herberts P, Hultmark P, Malchau H, Nivbrant B, Thanner J. Radiostereometry of hip prostheses. Review of methodology and clinical results. Clin Orthop Relat Res. 1997;344:94–110. 9372762

[46] Kaptein BL, Valstar ER, Stoel BC, Rozing PM, Reiber JH. A new type of model-based Roentgen stereophotogrammetric analysis for solving the occluded marker problem. J Biomech. 2005;38(11):2330–4. 16154422 10.1016/j.jbiomech.2004.09.018

[47] Kruijntjens D, Koster L, Kaptein BL, Jutten LMC, Arts JJ, Ten Broeke RHM. Early stabilization of the uncemented Symax hip stem in a 2-year RSA study. Acta Orthop. 2020;91(2):159–64. 31928120 10.1080/17453674.2019.1709956 PMC7144261

[48] Hartman CW, Gilbert BJ, Paprosky WG. Gender issues in total hip arthroplasty: length, offset, and osteoporosis. Seminars in Arthroplasty. 2009;20(1):62–5.

